# Osteosarcoma: A comprehensive review of model systems and experimental therapies

**DOI:** 10.18103/mra.v12i11.6000

**Published:** 2024-11-29

**Authors:** Gabrielle M. Robbins, Young Y. Vue, Eric P. Rahrmann, Branden S. Moriarity

**Affiliations:** 1 Department of Pediatrics, University of Minnesota, Minneapolis, MN 55455, USA; 2 Masonic Cancer Center, University of Minnesota, Minneapolis, MN 55455, USA; 3 Center for Genome Engineering, University of Minnesota, Minneapolis, MN 55455, USA; 4 College of Veterinary Medicine, University of Minnesota, Saint Paul, MN 55455, USA; 5 The Hormel Institute, University of Minnesota, Austin, MN 55912, USA

## Abstract

Osteosarcoma (OSA) is a highly malignant bone tumor for which more than 50% of patients have or will develop metastatic disease, resulting in an abysmal 5-year survival rate of <29%. Despite the advances in science and medicine, the etiology of OSA remains unclear. Similarly, the standard of care (surgery and chemotherapy) has changed little in the past 5 decades. This stagnation in treatment options is in part due to inadequate preclinical models for OSA; many of these models are oversimplified and do not account for the complexities of patient disease. Further, current treatments are harsh and invasive (e.g. high dose chemotherapy and potential limb removal) leading to a reduction in a patient’s quality of life (e.g. hearing loss, infertility, neuropathy), highlighting a need for developing more effective treatment strategies. Many experimental therapies have been tested in the preclinical and preclinical setting, with varying degrees of success. In this review, we will focus on pediatric and adolescent OSA, highlighting current animal models and experimental therapies.

## II. Introduction

The term “osteosarcoma” was first coined by French surgeon, Alexis Boyer, in 1805 to describe osseous tumors he observed during his practice^1,2^. Research focused on the cellular origins of OSA have identified osteoblasts, bone-forming progenitor cells, as a population of cells that give rise to OSA^3^. Though osteoblasts are a common cell of origin for OSA, the disease has a high degree of heterogeneity not only at the molecular level but also at the clinical level. Observations in the disproportionate presentation of OSA in the patient population have been correlated with skeletal structure size, which in part explains higher occurrences in males to females 1.2:1 and people of African origin compared to other ethnicities^4,5^. Further, age is a large risk factor for OSA formation. OSA has a bimodal distribution with 87% of cases during adolescence (0–24 years of age with 8.6 cases per million) and a second, smaller peak in elderly populations (>60 years of age with 2 cases per million), which only accounts for 13% of all osteosarcoma cases^5–10^.

Despite the many risk factors for developing OSA, this cancer develops fairly predictably in children and adolescents with the most common site being near the metaphyseal growth plates of long bones of the limbs: femur and tibia at 42% and 19%, respectively, with 75% of tumors in the femur occurring in distally and 80% of tumors in the tibia occurring proximally^6,8^. The location and size of the primary tumor highly impacts outcome. Patients with tumors located on the axial skeleton, femur, or the trunk of the body have a decreased survival time of 10 years compared to patients with tumors located on the appendicular skeleton (e.g. humerus and tibia)^11,12^.

The current standard of care for OSA, which has remained unchanged since the 1970s (neoadjuvant chemotherapy, tumor resection, adjuvant chemotherapy) is harsh and invasive with a 5-year survival of ~68%^7,8,13^. Current surgical procedures for OSA favor limb salvage, a highly invasive surgery to remove the tumor in an effort to preserve the major functions of the limb and carries high risks of mobility impairment and nerve damage. In cases where the limb cannot be saved, amputation–the previous gold standard, is performed to remove the tumor^13,14^. However, the efficacy of these approaches drastically declines based on the size of tumors. Bulkier tumors (>15cm diameter) have a 3.4 times greater morbidity risk due to challenges of limb salvage surgery, poorer response to chemotherapy, and greater probability of recurrence^11^. Overall, males are less responsive to chemotherapy with a higher tendency of recurrence while females correlate with better response to chemotherapy having an overall greater survival of about 16 months^11,15,16^. The highest rates of morbidity are attributed to the presence of metastatic disease. Metastasis of OSA occurs in the lung in >90% of documented cases while the next most common site of metastasis being other bones in 5–10% of documented cases^17,18^. At initial presentation, overt metastases are detected in ~25% of patients, which is correlated to poor prognosis^4^. Unfortunately, most patients are presumed to have subclinical micrometastatic lesions at the time of diagnosis^19^. Genetic drivers and predisposition syndromes to OSA have been reviewed extensively by *Beird et al*^10^.

**Figure 1.**
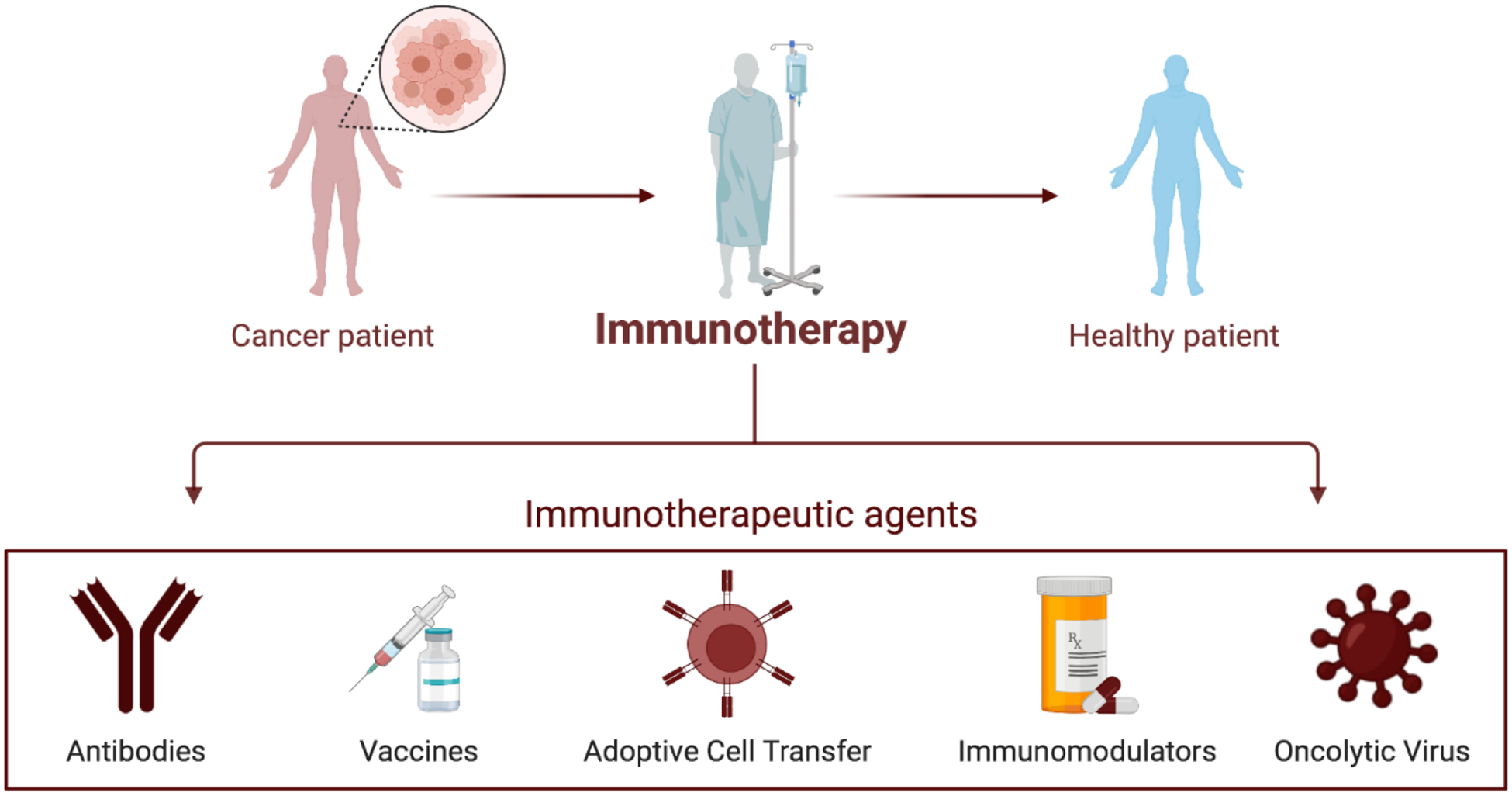
Experimental immunotherapies for cancer. A schematic detailing current common immunotherapy methods used to treat patients with cancer.

Despite advances in our understanding of OSA tumor biology, patient outcome remains poor, particularly with the presence of metastatic disease. Novel experimental treatments utilizing immunotherapeutic agents is a promising field of research with the potential to provide non-invasive and specific treatments for OSA. These treatments are highlighted in Figure 1. In this review, we will specifically focus on highlighting model systems and experimental therapies for the treatment of pediatric OSA.

## III. *In vitro* and *in vivo* Model Systems

*In vitro* based tumor models range in their complexity and are used to provide insight into tumor genetics, growth/proliferation, migration/invasion, and response to experimental therapies. As we advance our understanding of tumor cell biology, the complexity of these models has developed rapidly. *In vitro* based models include cell line-based models, induced pluripotent stem cell-derived models, and three dimensional (3D) models. *In vitro* models have been summarized in table 1.

Animal models are a useful tool to understand the genetic basis of OSA and, more importantly, to advance preclinical studies for the generation of new therapeutic approaches. Animal models that accurately recapitulate the natural course of disease are the most informative, however, etiology and pathogenesis of OSA remain poorly understood. Therefore, establishment and induction of representative animal models remains challenging and incomplete. To date, mice are the most used species to generate OSA models, however, spontaneous cases of OSA in canines represent an additional relevant and validated model of OSA. These animal models are outlined in table 2.

## IV. Experimental Therapies

### GENE THERAPY

Gene therapy is broadly defined as the transfer of genetic materials (DNA, RNA) into cells for the treatment of disease^57^. The genomic complexity and high mutational burden observed in OSA tumors has made gene therapies an attractive approach for prevention and treatment^58^.

Early attempts to treat OSA using gene therapy-based approaches focused on restoring function to commonly lost tumor suppressor genes (namely, *TP53* and *RB*). TP53 is a multifunctional protein effectively involved in all of the hallmarks of cancer and has been found to be mutated in 9.5% of OSA patients^59,60^. *Phelan et al* showed that delivery of a functional TP53 using the herpes protein VP22 resulted in OSA cell lines regaining the ability to induce apoptosis^61^. *Densmore et al* evaluated a murine OSA metastasis model with the treatment of P53 plasmid DNA, which showed a significant decrease in the number of tumor nodules and the size of the nodules that did form^62^. Several studies have shown that restoring or overexpressing P53 results in an increased sensitivity to chemotherapy drugs^63–66^. RB is involved in cell cycle control by binding E2F family transcription factors until being phosphorylated by the CDK4/cyclin-D complex and is inactivated in ~50% of OSA tumors^67,68^. Despite the frequency of *RB* mutations in OSA patients, *RB* heterozygous mice do not develop OSA and *RB* knockout (KO) is lethal^69–71^. While *RB* mutations do not appear to be causative of OSA, loss of heterozygosity (LOH) at the *RB* locus is common in high-grade tumors and is associated with poor patient prognosis^72,73^. Several studies have aimed to restore function to the *RB* pathway using a replication-deficient recombinant adenovirus vector and showed a decrease in tumorigenicity *in vivo*^74,75^. However, this approach was less effective in established xenograft models.

Beyond tumor suppressor genes, there are other genomic alterations that result in overexpression or upregulation; these oncogenes have been targeted for suppression for the treatment of OSA and are summarized in Table 3.

Several studies have utilized gene therapy approaches that are less dependent on patient tumor genetics to provide a more generalized therapy; approaches include introduction of IL-12, B7–1 gene transfer, herpes simplex thymidine kinase (HSV-TK), among others^105–109^. While *in vivo* results were promising, these models used OSA cells transduced prior to injection, which is not representative of treatment administered in a clinical setting. A major challenge for gene therapy-based treatments is that each OSA patient has unique genetics and mutational burdens. Despite some initial, promising success, a major challenge facing gene therapy based precision medicine for OSA is that the most frequent genomic alterations (namely, TP53 and RB) are challenging to target therapeutically and *in vitro* success has not translated to the patient setting^110^. More universally applicable gene therapy approaches are critical to improving patient outcome, particularly for metastatic disease. One such target may be forkhead box P1 (FOXP1), which in OSA acts as an oncogene by suppressing the TP53-P21-RB cascade^111^. Other considerations to make gene therapy a more feasible approach to treat OSA include efforts to decrease the cost and time to develop these therapies.

### SMALL MOLECULES INHIBITORS

Since the dawn of modern cell biology in the 1980s, small molecule inhibitors have become one of the primary methods for targeted cancer treatment^112,113^. Small molecule inhibitors work by interrupting target proteins’ function by binding to these proteins or their receptors^114^. When compared to other cancer therapies, small molecule inhibitors have several advantages, including binding a wider range of targets due to their small size, ability to be taken orally, and ability to penetrate the blood-brain barrier^115^. The majority of small molecule inhibitors target protein kinases, which catalyze the transfer of the phosphate group in ATP to the hydroxyl group of substrate proteins^116^. Small molecule inhibitors tested for OSA are summarized in Table 4.

Despite success in other tumor types and *in vitro* and *in vivo* in OSA models, there are still many challenges facing small molecule inhibitors. Success has been limited and further investigation into safety, low response rates, patient-patient genetic variation, and tumor resistance is critical to making small molecule inhibitors more effective for the treatment of OSA.

### IMMUNOTHERAPY

**Figure 2.**
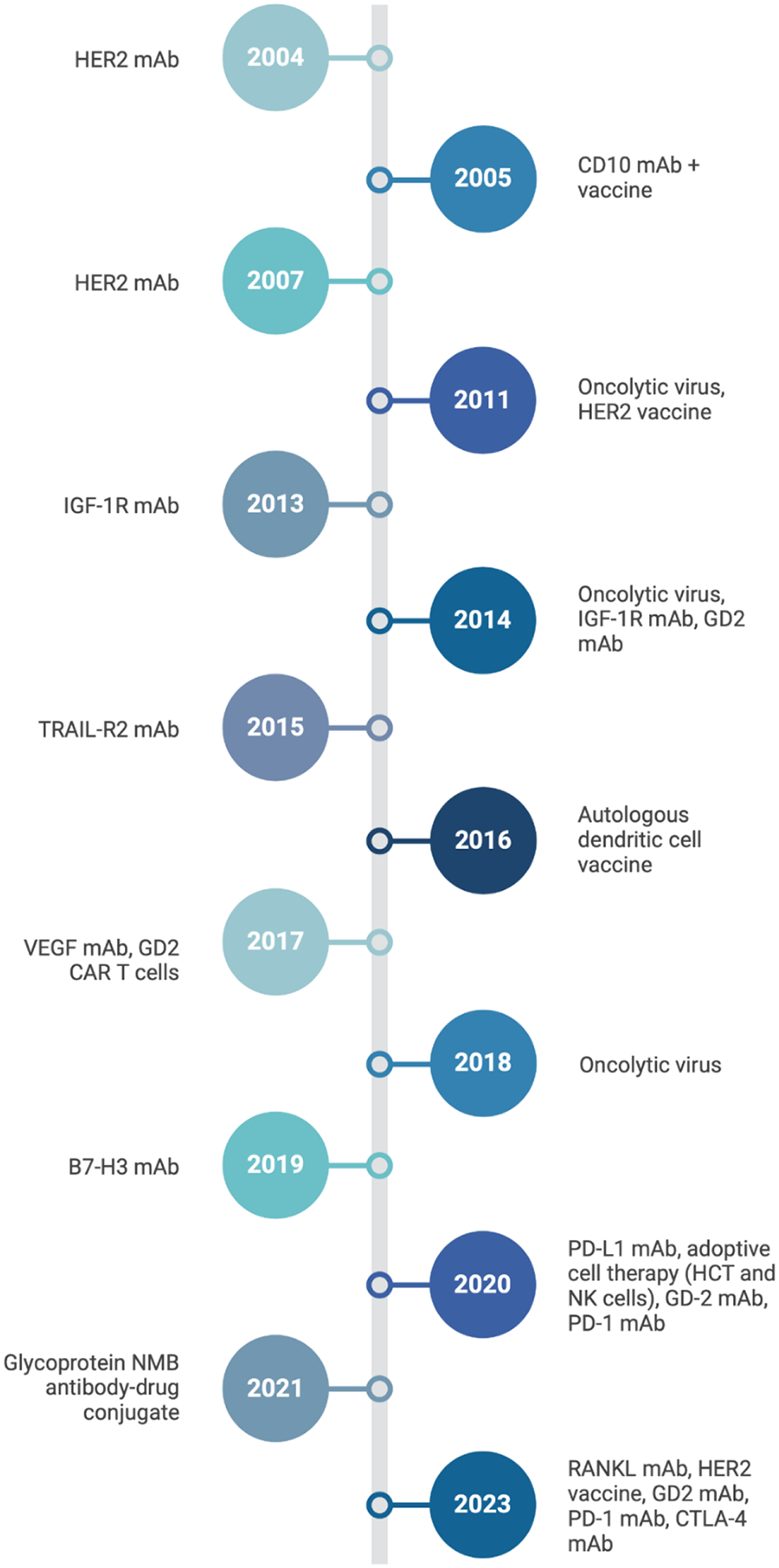
Timeline of osteosarcoma treatment and therapeutic development. Timeline showing development of immunotherapies for OSA.

Since William Coley, the father of immunotherapy, first tried to harness the immune system in 19th century bone cancer patients, immune-based therapies have drastically transformed cancer research and patient care^166^. Presently, cancer immunotherapies function by aiding the body in the detection and/or elimination of cancer cells^167^. Immunotherapy based approaches to treat cancer include monoclonal antibodies, cancer vaccines, and adoptive cell therapy. The timeline of development, type, and target of immunotherapy for OSA is showcased in Figure 2.

#### MONOCLONAL ANTIBODIES

Antibodies are glycoproteins in the immunoglobulin superfamily that recognize and neutralize foreign antigens while initiating an immune response^168^. Monoclonal antibodies (mAbs) are antibodies produced by individual B cell clones and specifically recognize a single antigenic determinant, or ‘epitope’^169^. A single pathogen induces the production of numerous antibodies targeting different epitopes found on that pathogen. Targeted monoclonal antibodies induce tumor cell death through a variety of mechanisms, including blocking critical tumor cell receptors or ligands, antibody-dependent cellular cytotoxicity (ADCC), antibody-dependent cellular phagocytosis (ADCP), or complement dependent cytotoxicity (CDC)^170^.

Thus far, monoclonal antibodies have not provided a breakthrough in the treatment of OSA. Many monoclonal antibody treatments have shown underwhelming results in clinical trials with limited efficacy (Table 5). However, they have led to the understanding of many important pathways and tumor response mechanisms that will be critical in identifying cures. A preclinical study using the K7M2 model assessed a combination CTLA-4 and PD-1/PD-L1 blockade and showed promise and may help patients where PD-1/PD-L1 blockade alone is not sufficient due to tumor escape^211^. SEMA4D has emerged as another promising target in the semaphorin family, especially due to signaling through its receptors PLXNB1 and PLXNB2, which regulates cell migration, survival, and tumor vascularization^212,213^. Monoclonal antibodies may serve an important role in preventing metastatic disease, the primary indicator of poor patient outcome for OSA.

#### CANCER VACCINES

Immune escape is one of the hallmarks of cancer. To resensitize the immune system of a patient, cancer vaccines aim to stimulate anti-tumor immunity through the presentation of tumor antigens^214^. One group used an attenuated *Salmonella typhimurium* vaccine to augment innate immune responses^215^. They achieved a partial response in one of the four canine OSA patients treated, a modest effect. *Hashii et al* evaluated a vaccine targeting Wilms tumor gene 1 (WT1), which is overexpressed in many pediatric cancers^216^. Unfortunately, the single OSA patient in this study did not benefit. *Himoudi et al* tested an autologous dendritic cell vaccination against OSA in a phase I clinical trial^217^. Of the twelve OSA cases, only two patients showed strong, T-cell immune responses while another patient had a strong, but non-specific immune response. *Finocchiaro et al* evaluated the vaccination of canine OSA cell lines with a vaccine containing cytokine-producing cells (human GM-CSF and human IL-2)^218^. Preclinical *in vitro* experiments showed promise and early clinical trial data shows a partial response in one canine patient and stable disease in two of the five canine patients that have undergone treatment. *Gentschev et al* showed potent effects of an oncolytic vaccinia virus against canine OSA *in vitro*^219^. Recently, *Cascini et al* used a TLR9 agonist that effectively activated an innate and adaptive immune response, indicating this agonist acted as an *in situ* vaccine against OSA^220^. *Mason et al* have reported on a recombinant Listeria monocytogenes vaccine that expresses a chimeric human HER2/neu construct^221^. In spontaneous canine OSA, this HER2 listeria vaccine inhibited lung metastasis and prolonged survival. As we continue to explore novel therapies for OSA, it is becoming increasingly apparent that combination therapies are critical, raising a number of questions. How will cancer vaccines fit into this picture? Can we develop vaccines that will sensitize the tumor to the patient’s immune system to prevent metastatic disease or even sensitive the tumor to additional chemotherapy or adoptive cell therapy?

#### ADOPTIVE CELL THERAPY

Over the past few decades, harnessing the immune system has become an attractive area of research for the treatment of cancer. Although targeted immunotherapies, such as monoclonal antibodies and cancer vaccines, have been efficacious in improving survival in some tumor types, solid tumors remain challenging. Adoptive cell therapy is another immunotherapy that has gained significant attention and is rapidly evolving. This cell-based therapy involves isolating patient or healthy donor immune cells, expanding them with or without modifications, and then administering these to the patient to mount an antitumor response. There are three sources of cells currently being developed for use as cell-based therapies; these include autologous, allogeneic, and xenogeneic cells^222^. Autologous cells are derived from the patient, allogeneic cells are human but from a healthy donor (not the patient), and xenogeneic cells are of non-human origin. Adoptive cell therapies can be grouped into three primary types, these include tumor-infiltrating lymphocytes (TILs), T cell receptor (TCR) therapy, and chimeric antigen receptors (CARs).

##### TUMOR-INFILTRATING LYMPHOCYTES.

Tumor-infiltrating lymphocytes (TILs) are detected in >75% of OSAs and include T cells, B cells, NK cells, macrophage, and mast cells^223^. What makes TILs unique is they are considered to have higher specific reactivity against tumors when compared to normal lymphocytes^224^. Unfortunately, patient data suggests that OSA is largely an immunologically ‘cold’ tumor that lacks tumor neoantigens and immune cell infiltration^225^. Traditionally, TILs are harvested from resected tumors, expanded *in vitro*, and then administered to patients^226^. As of recent years, TIL therapies only utilize T cells^227^. *Casanova et al* showed that in OSA, the presence of TILs was correlated with a better prognosis, supporting further investigation of TIL adoptive cell therapies for the treatment of OSA^228^. *Zhou et al* performed a retrospective analysis of adoptive TIL therapy plus the addition of anti-PD1 in patients with metastatic OSA^229^. They found that patients given this combination therapy exhibited increased progression free survival and overall survival. *Wang et al* reported on the successful isolation and expansion of TILs and the subsequent administration of these cells with anti-PD1 therapy^230^. Similar to the retrospective study, they found that the combination therapy of TILs and anti-PD1 resulted in improved patient outcome. As is evident by the dearth of publications, strategies for TIL isolation from OSA and subsequent expansion are not yet optimized, resulting in inadequate cell numbers to be administered therapeutically. Another limitation of TIL based adoptive cell therapies is the immunosuppressive nature of OSA. OSA is known to support a very immunoregulatory and inhibitory microenvironment, which could prevent the activation of TILs^231^.

##### T CELL RECEPTOR THERAPY.

Building on the concepts of TILs, T cell receptor (TCR) therapy uses engineered T cells to express a novel TCR for the recognition of tumor associated antigen (TAA)^232^. T cells are typically transduced using viral based methods, however, non-viral systems, such as transposons and CRISPR/Cas9, are being developed^233^. *Watanabe et al* generated a TCR multimer with high avidity for naturally occurring TAAs on OSA cells^234^. While TCR therapies have shown promise in other tumor types, the field remains largely unexplored with respect to OSA. Due to the genomic instability and variability often seen between patients, identification of individual patient neoantigens will be cost and labor intensive. Another concern, especially with an immunologically cold tumor like OSA, is ensuring that these TCR engineered T cells traffic to the tumor. Further, a major limitation of all T cell-based therapies is that they are HLA restricted and thus must be HLA matched to be effective and safer for the patient. Success of TCR-based adoptive cell therapies will be largely dependent on improving peptide MHC-TCR interactions and identifying shared TAAs^235^.

##### CHIMERIC ANTIGEN RECEPTORS.

CARs are engineered receptors designed to graft immune effector cells with the ability to specifically target TAAs independent of MHC restriction and activate modified cells through signal transduction^236^. Historically, most studies have evaluated CARs in T cells for the treatment of various cancers, however, other immune cell types are emerging as attractive effector cells, such as NK, macrophage, and others^224^. CAR-T cell therapies have been very successful in the treatment of hematological cancers, which has sparked interest in designing new CARs for the treatment of more diverse malignancies, including solid tumors^237^. CAR constructs are composed of an extracellular binding domain known as the single-chain fragment variable (scFv), a hinge region, transmembrane domain, intracellular signaling domains, and costimulatory domains^238^. Three CAR targets that have received notable attention for OSA include HER2, B7H3, and GD2. *Ahmed et al* evaluated HER2 CAR T cells against OSA, showing superior cytotoxicity *in vitro* and *in vivo*^239^. *Rainusso et al* showed similar results^240^. *Talbot et al* showed potent antitumor activity of B7H3 CAR T cells against OSA^241^. Similarly, *Zhang et al* also evaluated B7H3 CAR T cells showing anti-tumor activity against OSA *in vitro* and *in vivo*^242^. Interestingly, *Hidalgo et al* evaluated CAR T cells against, using B7H3 as a target and showed antitumor activity *in vitro* and *in vivo*^243^. Their strategy utilized intermediary switch molecules, which are adaptors that target TAAs and selectively bind the CAR, mediating interactions between CAR T cells and tumors allowing for improved safety. *Chulanetra et al* generated GD2 CAR T cells, which showed effective killing of OSA *in vitro*^244^. Interestingly, OSA cells that survived treatment with GD2 CAR T cells upregulate PD-L1, suggesting that a combination therapy may be beneficial. *Fernández et al* evaluated NKG2D-CAR T cells against OSA, which showed increased cytotoxic activity *in vitro* and *in vivo*^245^. *Huang et al* tested interleukin (IL)-11 Rα CAR T cells against OSA, showing a regression of pulmonary metastases in a mouse model^246^. *Wang et al* generated CD166 CAR T cells, which showed antigen expression dependent cytotoxicity against OSA *in vitro* and tumor regression *in vivo*^247^. CART cells targeting an isoform of alkaline phosphatase, ALPL-1, generated an effective anti-tumor response against OSA *in vitro* and *in vivo*^248^.

**Figure 3.**
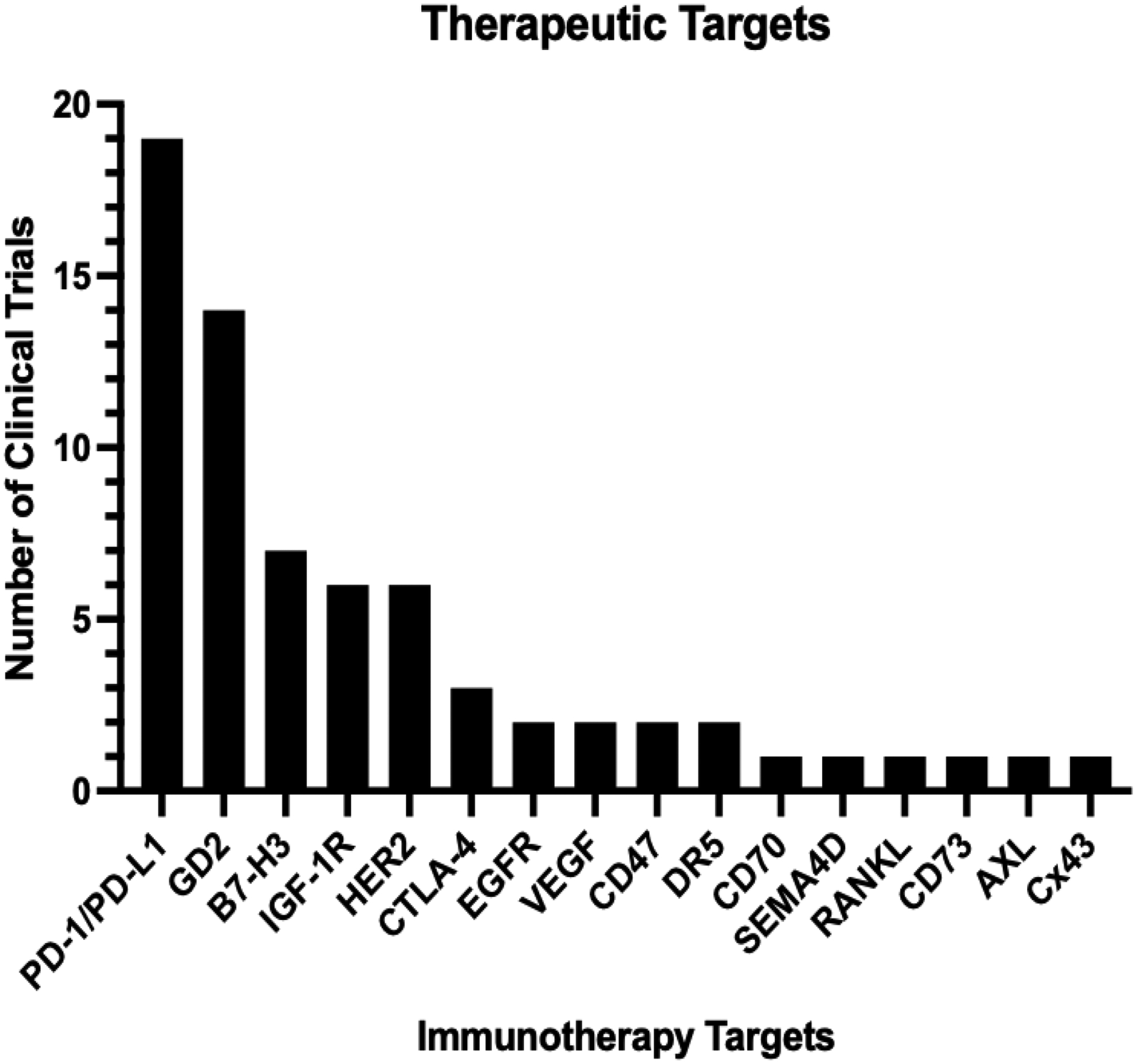
Osteosarcoma clinical trial therapeutic targets that have been tested. The bar graph shows the frequency of different targets in completed clinical trials.

To date, there have been many targets of CAR T cell therapies, and many have made it into clinical trials (Figure 3). To further improve the success of CAR T cell therapies, particularly against solid tumors, there are several limitations to address. First, these therapies are associated with several toxicities in patients, including cytokine release syndrome (CRS), prolonged cytopenias, neurological toxicity, among others^249^. Other limitations include antigen escape, limited persistence, poor homing/infiltration, and inactivation by immunosuppressive tumor microenvironments^250^.

## V. Conclusion

Over the last several decades, scientific efforts have advanced our understanding of osteosarcomagenesis, its genetic landscape, metastasis, prognostic markers, and the development of effective preclinical *in vitro* and *in vivo* model systems. New molecular techniques have allowed for the generation and exploration of novel targets and therapies. Unfortunately, patient outcome has remained stagnant for *five* decades, with a dismal prognosis for patients with relapsed and/or metastatic disease. As a rare tumor, human OSA research would benefit from continued interdisciplinary research through collaborations with veterinarians working with canine patients, a species that sees more than 25,000 cases of OSA annually. The variation between patients with respect to tumor antigen expression poses another major challenge for developing an all-encompassing therapy. However, some targets are more broadly expressed by OSAs, such as HER2, B7H3, and GD2. OSAs with greater immune infiltration hold a better prognosis than their typical ‘cold’ counterparts.

**Figure 4.**
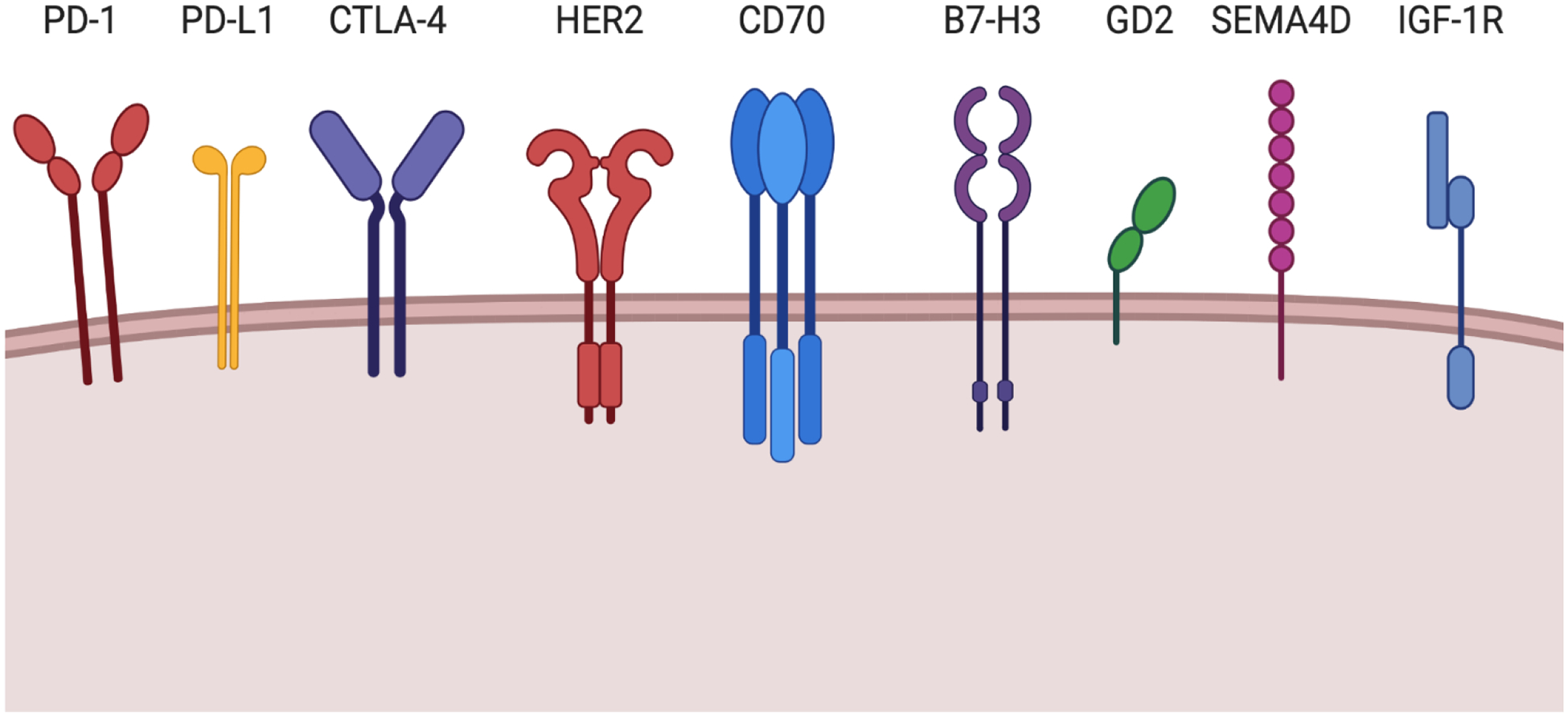
Promising immunotherapy targets of interest for osteosarcoma focused CAR-therapy. Graphical schematic showing surface proteins that show promise for future therapeutic targeting for OSA.

Cell-based therapies hold great promise in resensitizing these tumors to immune activity. Novel immunotherapy targets, such as Ephrin-A2^251^, are critically needed. To date, all OSA CAR therapy publications have utilized T cells, however, many other immune cell types hold promise as potential effector cells, including natural killer (NK) cells, *γ*Δ T cells, and monocytes/macrophage. These other immune effector cells overcome several limitations of CART cells, notably patient toxicities. However, cell-based and other immunotherapies are limited by tumor immune escape, something commonly seen with OSA. Developing immunotherapies to target multiple antigens and/or expressing factors to promote their sustainability would undoubtedly enhance success in the treatment of OSA. Current targets of interest for immunotherapy against osteosarcoma are shown in Figure 4. Finally, combination therapy utilizing multiple targets will likely be critical to prolonged tumor control in relapsed and/or metastatic patients.

## Figures and Tables

**Table 1.** *In vitro* models of osteosarcoma. Methods of use, advantages, and disadvantages of each model are highlighted.

Model	Methods / Relevant Findings	Advantages	Disadvantages	References
**Cell line based**	Adherent 2D cultures Monolayer Cell lines used include 143B, HOS, U2OS, SJSA-1, and G-292, among others	Widely established Low-cost maintenance Ease of culturing Usability in various functional assays	Do not recapitulate the natural structure or microenvironment seen in patient tumors Cannot mimic complex interactions between tumor cells, other cell types, and stimuli found in the tumor microenvironment Altered response to experimental therapies Changes in morphology, gene expression, signaling, polarity, topology, and loss of diverse phenotypes compared to original tumor tissue	^20,21^
**Induced pluripotent stem cell-derived**	TP53 and RB1 focused models Rothmund-Thomson syndrome (RTS) model showed link to elevated mitochondrial respiratory complex I function	Able to be maintained indefinitely in an undifferentiated, pluripotent state using Yamanaka factors (OCT3/4, SOX2, KLT4, cMYC) Ability to be differentiated into any human cell type Fast to generate	Limited success generating these models from malignant cells Engineering non-malignant cells to exhibit the genomic instability of OSA has not yet been achieved Not well understood how iPSC models mimic tumor heterogeneity Directed differentiation can result in a more immature phenotype	^22–30^
**Three dimensional (3D)**	Multicellular tumor spheroids (MCTSs) Aggregates of cells grown in a 3D matrix that mimic the physical and biochemical properties of a tumor Beyond MCTSs, 3D tumor bioprinting allows for the combination of cells, biomolecules, and biomaterials into into organized, complex structures that mimic the characteristics of bone Several MCTS and bioprinting models have been developed to study OS	Able to recapitulate critical elements of the tumor microenvironment, including cell-cell interactions Sustain oxygen and nutrient gradients that result in necrotic cores seen in many tumor types Protein and gene expression profiles mirror patient data more closely than 2D models	Expensive Time consuming to generate 3D modeling is in its relative infancy with more limited publications	^31–44^

**Table 2.** Methods of establishing animal models. Different *in vivo* model systems are listed with accompanying descriptions, immune competency of these models, as well as metastatic potential. All included methods for generating *in vivo* models have metastatic potential, however, some models may be transgene or cell line dependent.

Model Type	Description	Immune Component	Metastatic	References
**Transgenics**	De *novo* formation is induced by Cre-*loxP*-mediated inactivation of TSG alleles and/or activation of conditional oncogenes. Tissue specific expression of the Cre-recombinase is achieved through crossbreeding with Cre transgenic mice, tamoxifen-inducible Cre-ERT transgenic mice, or by local administration of Cre-encoding lenti- or adenoviruses	Yes	Yes	^45^
**Allografts**	LM8 derived by collecting OSA lung metastases derived from the Dunn-cell line-derived model K7M2 derived from collecting lung metastases following intraosseous injection of K7 cell line (spontaneous murine OS)	Yes	Yes	^46–48^
**Xenografts**	143B is derived from a 13-year-old patient’s tumor HOSA is derived from a 13-year-old primary patient’s tumor. Has mutations in *CDKN2A* and *TP53*. U2OSA is derived from a 15-year-old patient’s primary. No mutations detected in 64+ commonly mutated genes. SaOS-2 is derived from an 11-year-old patient’s primary tumor. Has mutations in *TP53* and *RB1*. Expresses TGF-beta type 1 and 2. MG-63 is derived from a 14-year-old patient’s tumor. Expresses high levels of TGF-beta.	No	Yes	^49–53^
**PDXs**	Generated using immunocompromised mice implanted subcutaneously with patient derived tissue	No	Yes	^54^
**Canine**	Greater incidence in large and giant breed dogs, including Rottweilers, German Shepherds, Boxers, and Dobermans Most frequent in middle-aged dogs between the ages of 6–10, however, smaller secondary peak in dogs between the ages of 1–2 Similar genetics to human patients; canine OSA is not inherited	Yes	Yes	^55,56^

**Table 3.** Summarized gene therapy targets. Oncogenes targeted for suppression for the treatment of OSA with variable degrees of efficacy during *in vitro* and *in vivo* testing.

Gene Therapy Target	Findings in OS	References
**Vascular Endothelial Growth Factor (VEGF)**	Pro-angiogenic factors associated with tumor angiogenesis Silencing VEGF using siRNAs suppressed tumor growth, reduced angiogenesis, and downregulation of PI3K and AKT *in vivo*	^76–78^
**Apurinic/Apyrimidinic Endonuclease 1 (APE1)**	Plays a critical role in DNA repair and redox regulation *APE1* has been shown to be overexpressed in OSA and is associated with chemoresistance and poor patient outcome miRNAs silencing of *APE1* demonstrated the function of APE1 in inhibiting DNA damage repair and sensitization of OSA cells to cisplatin Silencing *APE1* expression showed anti-angiogenic effects, increased apoptosis, and VEGF suppression in OSA xenografts	^79–83^
**Insulin-Like Growth Factor 1 (IGF1R)**	14% of OSA tumors have amplification of IGF1R miRNA targeting of IGF1R inhibited activation of AKT and ERK signaling pathways	^84,85^
**c-Jun**	High grade OSAs show an increase in the transcription factor c-Jun c-Jun DNAzyme inhibited OSA growth and metastasis *in vitro* and *in vivo* in an orthotopic OSA model miRNA targeting of Hsp70 to modulate downstream JNK/JUN signaling pathway modulated OSA chemoresistance	^86–89^
**Ezrin**	Metastasis-associated Ezrin functions in a cancer setting by allowing cells to overcome several stresses during the metastatic cascade, including initiating the translation of new proteins and efficient ATP generation OSA patients with lung metastases have shown a fivefold increase in *Ezrin* mRNA expression when compared to non-metastatic patients *In* vitro use of Ezrin-specific siRNA showed significant reduction in growth rate and cell morphology	^90–93^
**Cyclin A2 (CCNA2)**	CCNA2 is a critical regulator of cell division that has been shown to be overexpressed in OSA and is linked to poor patient prognosis and metastasis Knock down of CCNA2 using siRNAs showed dramatic decreases in proliferation *in vitro* miRNA repression of CCNA2 inhibited proliferation, migration, and colony-formation of aggressive OSA cell lines	^94–97^
**Urokinase plasminogen activator and receptor (uPA/uPAR)**	uPA and uPAR play critical roles in tumor invasion, migration, angiogenesis, and metastasis Expression of uPA and uPAR have been reported in human and canine OSA with an association between enhanced metastatic behavior Anti-uPAR DNAzymes showed a decrease in uPAR transcript by over 80%, resulting in significantly decreased OSA cell invasion *in vitro* shRNA silencing of uPAR and small molecule inhibition of uPA showed decreased migratory response and decreased or total inhibition of metastasis of OSA *in vitro* and *in vivo*	^98–104^

**Table 4.** Summarized small molecule inhibitors. Each small molecule has one or many targets with variable degrees of efficacy against osteosarcoma during preclinical and clinical testing.

Small Molecule Inhibitor	Targets	Findings in OS	References
**Anlotinib**	VEGFR1–3, PDGFRα/β, FGFR1–4, Aurora B, EMT, RET, KIT	Suppresses tumor proliferation, angiogenesis, and metastasis *in vitro* and *in vivo*. Treatment increased chemo-sensitivity	^117–121^
**Apatinib**	VEGFR1–2, KIT, RET, v-src avian sarcoma viral oncogene homolog	Promotes apoptosis and autophagy while inhibiting invasion, migration, and PD-L1 expression *in vitro*. Phase II clinical trial showed a partial response rate of 43%	^122–125^
**Axitinib**	VEGFR1–3, PDGFRα/β, RET, KIT, FGFR1, CSF-1R	Children’s Oncology Group Phase I and pilot consortium trial showed 2 OSA patients presented with stable disease	^126–128^
**Cabozantinib**	VEGFR1–3, RET, KIT, FLT3, MET, PDGFRα/β	Decreases proliferation and migration *in vitro* by inhibiting ERK and AKT. Phase II clinical trial showed 12% of OSA patients had a partial response, 33% had 6-month stable disease	^129–132^
**Cediranib**	VEGFR1–3, KIT, FGFR1, PDGFRα/β	Showed no improvement in survival against OSA xenograft model Phase I clinical trial showed 1 of 4 OSA patients had a partial response	^133–136^
**Dasatinib**	BCR-ABL, SRC, LCK, YES, FYN, KIT, EPHA2, PDGFRβ	Evaluated in canine OS; 2 dogs showed stable disease to partial remission	^137,138^
**Fruquintinib**	VEGFR1–3	Improvement in proliferation free survival in retrospective study	^139,140^
**Imatinib**	PDGFRα/β, RYK, EGFR, EPHA2, EPHA10, IGF1R, KIT, BCR-ABL	Inhibited downstream signaling molecules such as AKT and ERK *in vitro*. Dose dependent anti-proliferative effect. This did not translate to *in vivo* mouse studies. Did not translate to the clinical setting	^141–145^
**Lenvatinib**	VEGFR1–3, FGFR1–4, PDGFRα, RET	No preclinical studies. Clinical trials have been underwhelming with few partial responders and a high incidence of serious treatment associated adverse events	^146–148^
**Nintedanib**	VEGFR1–3, FGFR1–2, PDGFRα/β	Increases apoptosis *in vitro* and blocked the formation of lung metastases *in vivo*	^149–151^
**Pazopanib**	VEGFR1–3, PDGFRα/β, KIT	*In vivo* combination treatment with chemotherapy drug topotecan reduced primary tumor growth. Retrospective study showed promising results of pazopanib treatment on 15 relapsed OSA patients	^152–154^
**Regorafenib**	VEGFR1–3, PDGFRα/β, KIT, FGFR1–2, RET, TEK	Treatment *in vitro* and *in vivo* induces apoptosis, reduces tumor growth, and reduces invasion. Findings did not translate to the clinical setting	^155–158^
**Sorfenib**	AXL, RAF, RGFR2, KIT, PDGFRα/β, RET	No effect of treatment on canine OSA cell viability *in vitro*. Phase II clinical trial had underwhelming results for patients with advanced or unresectable disease	^138,159,160^
**Sunitinib**	VEGFR1–3, KIT, FLT3, AXL, EPHB2, FGFR2, IGF1R, RET	Reduced tumor burden, tumor vasculature, and lung metastasis in mouse models of human OSA. Combination therapy even more effective	^161–165^

**Table 5.** Summary of many monoclonal antibody treatments for osteosarcoma. Each monoclonal antibody therapy has a single target with variable degrees of efficacy against osteosarcoma during preclinical and clinical testing.

Monoclonal Antibody	Target	Mechanism and Findings in OS	References
**Bevacizumab**	VEGF	Inhibit angiogenesis and thought to improve delivery of chemotherapy agents. Did not improve OSA patient outcome	^171–173^
**Ramucirumab**	VEGFR2	Dominant VEGF receptor responsible for mediating the functions of VEGF. Underwhelming results in murine models of OS	^174,175^
**Nivolumab**	PD-1	PD-L1/2 expression is negatively associated with patient outcome, recurrence, and metastasis in OS. *In vivo* treatment showed a decrease in the number of metastases, tumor apoptosis, decreased tumor cell proliferation, blockade of pSTAT3/pERK1/2 signaling, increased immune cell infiltration, and a decrease in pro-inflammatory M2 macrophages. Several clinical trials have been run to evaluate PD-1/PD-L1 blockage in OSA but limited success has been seen	^176–188^
**lpilimumab**	CTLA-4	CTLA-4 is expressed by OSA tumors and cell lines but preclinical investigations into CTLA-4 blockade in OSA are limited. Clinical trials targeting CTLA-4 have shown limited effect in OSA	^189–192^
**Anti-LRRC15**	LRRC15	LRRC15 is known to be tumorigenic and is overexpressed in OSA. It is also associated with increased incidence of metastasis, chemoresistance, and reduced patient survival. OSA cells high in LRRC15 expression showed inhibited growth when treated with anti-LRRC15. PDX models showed tumor growth inhibition. Phase I clinical trial evaluating anti-LRRC15 found an overall patient response rate of 20%	^193–196^
**Trastuzumab**	HER2	40–80% of OSAs express HER2 to varying degrees, with ~30% showing high levels of HER2. Due to the limited nature of anti-HER2 preclinical testing in OSA, success in other tumor types prompted a phase II clinical trial of trastuzumab in OSA. The outcome was poor for all patients involved in the study	^197–199^
**Anti-EGFR**	EGFR	EGFR has been found to be highly expressed in ~50% of OSA patients and while it is not a driver of tumor growth, it does contribute to chemoresistance. Treatment with anti-EGFR potentiated and directed NK cell activity toward OSA cells *in vitro*	^200–203^
**Teprotumumab**	IGF-1R	IGF-1R is a glycoprotein overexpressed in OSA, aiding in tumor progression through transformation, proliferation, chemotherapy resistance, and metastasis. Preclinical testing showed promise in OSA with decreased tumor growth, increased event-free survival, and decreased AKT signaling. Phase I and II clinical trials evaluating cixutumumab (anti-IGF-1R mAb) in solid tumors showed limited efficacy	^204–207^
**Glembatumumab**	gpNMB	Preclinical studies showed high gpNMB expression on OSA cell lines and potent anti-tumor effects. A phase II clinical trial looking at glembatumumab in 22 relapsed or refractory OSA patients showed only one patient with a partial response	^208,209^
**Anti-SEMA4C**	SEMA4C	SEMA4C was shown to be overexpressed and antibody targeting promoted adhesion while reducing proliferation, colony formation, migration, wound healing, tumor growth, and metastasis in OSA	^210^
